# Prognostic and therapeutic implications of *BRAF* mutations in acute myeloid leukemia

**DOI:** 10.1038/s41375-026-02996-1

**Published:** 2026-06-15

**Authors:** Darren Lee, Yazan Abu-Shihab, Kate Plas, Deedra Nicolet, Krzysztof Mrózek, Mark J. Routbort, Keyur P. Patel, Christopher J. Walker, Jill Buss, Andrew R. Stiff, Andrea Laganson, Courtney D. DiNardo, Naval G. Daver, Tapan M. Kadia, Farhad Ravandi, Andrew J. Carroll, Jonathan E. Kolitz, Bayard L. Powell, William G. Blum, Maria R. Baer, Guido Marcucci, Geoffrey L. Uy, Wendy Stock, Richard M. Stone, L. Jeffrey Medeiros, John C. Byrd, James S. Blachly, Robert L. Bowman, Jeffrey W. Tyner, Sanam Loghavi, Ann-Kathrin Eisfeld, Linde A. Miles

**Affiliations:** 1 University of Cincinnati College of Medicine, Cincinnati, OH, USA.; 2 Division of Hematology, Department of Internal Medicine, The Ohio State University Comprehensive Cancer Center, Columbus, OH, USA.; 3 University of Cincinnati, Computational Science Graduate Program, Cincinnati, OH, USA.; 4 Clara D. Bloomfield Center for Leukemia Outcomes Research, The Ohio State University Comprehensive Cancer Center, Columbus, OH, USA.; 5 Department of Hematopathology, MD Anderson Cancer Center, Houston, TX, USA.; 6 Department of Internal Medicine, Ohio State University, Columbus, OH, USA.; 7 Department of Leukemia, MD Anderson Cancer Center, Houston, TX, USA.; 8 Department of Genetics, University of Alabama at Birmingham, Birmingham, AL, USA.; 9 Northwell Cancer Center, Hofstra/Northwell School of Medicine, Lake Success, NY, USA.; 10 Atrium Health Wake Forest Baptist Comprehensive Cancer Center, Winston-Salem, NC, USA.; 11 Winship Cancer Institute of Emory University School of Medicine, Atlanta, GA, USA.; 12 University of Maryland Greenebaum Comprehensive Cancer Center, Baltimore, MD, USA.; 13 Department of Hematology, City of Hope Comprehensive Cancer, Duarte, CA, USA.; 14 Division of Oncology, Washington University School of Medicine, Saint Louis, MO, USA.; 15 University of Chicago Medicine, Chicago, IL, USA.; 16 Dana-Farber Cancer Institute, Boston, MA, USA.; 17 Department of Internal Medicine, University of Cincinnati, Cincinnati, OH, USA.; 18 University of Cincinnati Cancer Center, Cincinnati, OH, USA.; 19 Department of Cancer Biology, Perelman School of Medicine, University of Pennsylvania, Philadelphia, PA, USA.; 20 Department of Cell, Developmental & Cancer Biology, Knight Cancer Institute, Oregon Health & Sciences University, Portland, OR, USA.; 21 Division of Experimental Hematology & Cancer Biology, Cancer & Blood Diseases Institute, Cincinnati Children’s Hospital Medical Center, Cincinnati, OH, USA.; 22 Department of Pediatrics, University of Cincinnati, Cincinnati, OH, USA.

## Abstract

Mutations in the RAS/MAPK signaling pathway are recurrent in acute myeloid leukemia (AML), primarily involving *NRAS* and *KRAS*. In contrast, mutations in the gene encoding an effector protein, BRAF, occur at relatively lower frequencies in AML and are associated with poor outcomes. To date, no comprehensive analysis has assessed the clinical and molecular characteristics of *BRAF*-mutated AML. In this study, we report the identification of canonical and non-canonical *BRAF* mutations in ~1% of 5779 consecutive clinically and molecularly fully annotated AML patients treated at two major United States Cancer Centers (50/5779 AML patients: 21 newly diagnosed AML; 9 relapsed/refractory; 20 newly diagnosed secondary AML). We performed single-cell multiomic analysis on a subset of AML samples. *BRAF* mutations were enriched in myelodysplasia-related AML (AML-MR), and most mutations were located outside the V600 hotspot. Single-cell multiomic profiling delineated *BRAF* mutation class-specific patterns of co-mutations, clonality, and immunophenotypes. Notably, *BRAF* mutations and other signaling co-mutation(s) could be found in the same cell, a finding that significantly diverges from prior studies of *RAS*-mutant AML. In this cohort, *BRAF*-mutant AML patients had poor overall survival with currently available treatments, including venetoclax-based regimens. Drug sensitivity data suggest possible avenues for targeted treatment of *BRAF*-mutated AML.

## INTRODUCTION

Mutations in the RAS/MAPK pathway genes, including *NRAS, KRAS*, and *PTPN11*, occur in >20% of patients with acute myeloid leukemia (AML), both de novo and secondary [1, 2]. Typically, these mutations are subclonal, suggesting they are later, transforming events in disease development [3]. Mutations in *BRAF*, a gene encoding a protein kinase also in the RAS/MAPK signaling pathway, are well-known driver mutations in other cancer types [4, 5], such as melanoma [6], papillary thyroid cancer [4], colorectal cancer [4] and hairy cell leukemia [7]. *BRAF* mutations are also recurrent mutational events in AML, albeit at a much lower frequency [3, 8]. While RAS-mutant AML patients may respond to standard cytotoxic chemotherapy, depending on the molecular context, *BRAF* mutations in AML have been divergently associated with dismal outcomes [3]. However, since many routinely used genetic testing panels have limited *BRAF* gene coverage, *BRAF* mutations might be overlooked and, their actual frequency, especially in relapsed/refractory disease, is likely underestimated. Importantly, in view of the prognostic impact of RAS pathway mutations on non-intensive treatment regimens, there is an urgent need to better understand the impact of *BRAF* mutations in AML, including their clinical, molecular, and cellular context, as well as their associations with response to intensive and non-intensive treatment regimens.

The most common *BRAF* mutation in cancer, known as p.V600E, substitutes the valine residue at codon 600 with glutamic acid [8]. Some hematologic cancers, such as hairy cell leukemia, have recurrent *BRAF V600* mutations in ~100% of cases [7]. Notably, alternative non-canonical *BRAF* mutations exist in other cancer types and have been sorted separately from the V600E hotspot into two classes based on kinase activity and oncogenic RAS-dependence [5]. In total, three classes of *BRAF* mutations exist – class I (i.e. V600E) and class II mutations generate constitutively active kinases, but the class I mutations are unique in that these kinases can function as monomers [8-10]. In contrast, class II mutant BRAF kinases still require dimerization for activity. Conversely, class III mutations are kinase-dead mutants with little to no BRAF kinase activity [11, 12]. However, class III mutant BRAF proteins are still able to amplify RAS pathway signals, but require an active or oncogenic RAS to transmit activation of the pathway. Little is currently known about the impact of the various classes of *BRAF* mutations on AML disease phenotype and therapeutic consequences, which may be attributed to the rarity of these mutations compared with other recurrent AML-associated mutations. However, the actual frequency of *BRAF* mutations is unknown, because most routinely used targeted next-generation sequencing panels cover only the V600 *BRAF* mutation.

In this study, we identified a cohort of *BRAF*-mutant AML patients and examined their leukemic molecular landscape, clonal architecture, and response to therapy. We found that *BRAF* mutations were highly enriched in myelodysplasia-related AML (AML-MR) and that most of them were non-V600E, non-canonical *BRAF* mutations, with each class of *BRAF* mutation showing a unique co-mutation and immunophenotype pattern. Moreover, we found that *BRAF*-mutant AML patients had dismal outcomes, and their disease showed resistance to venetoclax similar to *RAS*-mutated AML. However, through characterization of published ex vivo drug treatment data, we also identified unique drug sensitivities for *BRAF*-mutant AML that are not shared by other RAS-pathway mutant AML.

## METHODS

### Patient cohort

Diagnosis according to World Health Organization (WHO) classification [13] and the International Consensus Classification (ICC) [14] criteria and disease status assignment are provided in Supplementary Table 1. Mononuclear cells were isolated via Ficoll gradient centrifugation from obtained bone marrow aspirates or peripheral blood specimens. Single-cell suspensions of samples were viably frozen and stored in liquid nitrogen.

### Bulk DNA molecular profiling

All patients and samples included in the *BRAF*-mutant clinical cohort and control cohorts underwent bulk DNA molecular profiling. Samples in the BeatAML cohort obtained from OHSU were sequenced with a combination of whole-exome sequencing, targeted sequencing panels, and clinical genetic platforms (Sequenom and GeneTrails [2, 15]). Analysis of samples collected at MDACC used targeted PCR-based NGS panels covering 81 recurrently mutated genes [16] or 28 recurrently mutated genes [17] in myeloid disorders. The Alliance samples were sequenced using an NGS panel covering 80 cancer and/or leukemia-associated genes, as described previously [18]. OSU patient sequencing was obtained from the patients’ clinical records using the OSU CLIA-approved panels.

### Whole-exome sequencing

Whole-exome hybrid capture and NGS sequencing were performed on a subset of *BRAF*-mutant AML samples at OSU. The capture included chromosomal tiling probes (250-fold coverage) and was performed on leukemic and matched germline tissue for each patient for the detection of single nucleotide variants and small insertions/deletions, as described previously [19].

### Reagents

Mission Bio Tapestri-related reagents were included in the Myeloid DNA +Protein V2 sequencing kit except: Cell Staining Buffer, TotalSeqD CD135 antibody (313325), and TotalSeqD Human Heme Oncology Cocktail v1 (399906) were purchased from Biolegend. The Myeloid amplicon panel is a previously described panel [20].

### Single-cell multi-omic sequencing

AML patient samples were thawed, washed with FACS buffer (PBS supplemented with 2% fetal bovine serum), filtered to obtain single-cell suspensions, and quantified using a CellDrop (Denovix). Cells (1 × 10^6^ viable cells) were then incubated with Tapestri blocking buffer and TruStainFcX (Biolegend) on ice for 15 min followed by a 30 min incubation with the TotalSeqD Antibody Cocktail supplemented with 2 μL of TotalSeqD CD135 on ice. Stained cells were then further processed as previously described in commercially available protocols [21-23]. Sequencing of pooled libraries were performed by the CCHMC DNA Genomic Sequencing shared facility.

### Single-cell DNA sequencing analysis

Sequencing reads were processed as follows: reads were trimmed, aligned (hg19 human genome), assigned barcodes, and genotypes called using GATK by the Mission Bio Tapestri v2 pipeline. Further analysis of processed H5 files was performed using the scDNA package (https://github.com/bowmanr/scDNA, v1.01) in R. In the scDNA package, H5 files were input and variants of interest were identified based on clinical sequencing identified variants. All variants included were manually investigated in IGV. We selected non-synonymous, exonic variants that were genotyped in >50% of assayed cells and had a computed VAF > 1%. The variant list was further refined to exclude variants that were 1) confirmed SNPs, 2) recurrently mutated broadly across cohorts at a fixed VAF, and/or 3) represented exclusively in low-quality reads or clipped reads. The ‘tapestri_h5_to_sce’ function from the scDNA package was used after variant selection to create a SingleCellExperiment class object using the default cutoffs of allele frequency (AF) variance >25, depth (DP) > 10, and genotype quality (GQ) > 30. The AF variance refers to the maximum deviation from 50% by which a heterozygous call should be masked as inaccurate. We only retained variants that passed all three filters in over 80% of cells and only cells that passed all three filters were included in the final analysis and termed “Complete” cells, indicating they received a reliable genotype for all genes of interest. Clones were identified and statistically summarized following variant identification using the ‘enumerate_clones’ and ‘compute_clone_statistics’ functions, respectively.

### Single cell DNA+Protein (scDNA+Protein) sequencing analysis

Protein matrices were extracted from the H5 files using the scDNA package after genotyping and clone enumeration above. The SingleCellExperiment object was converted to a Seurat object (v5.1) with metadata containing genotyping information. All complete cells identified above were bound to a single protein matrix for cohort level protein analysis across all samples. Protein data were normalized across cells using DSB, scaled across all samples, and analyzed by PCA. Samples were integrated with Harmony, clustered (SLM) and then subsequently visualized by Uniform Manifold Approximation and Projection (UMAP) [24]. Cell type calls were performed by manual interpretation of protein expression. Data were visualized using Seurat, ggplot2, gridExtra, ggpubr, magick, patchwork, raster and scCustomize packages (https://samuel-marsh.github.io/scCustomize/).

### Beat AML ex vivo drug screen data

Ex vivo functional drug screens were performed as described previously [2, 15] on freshly isolated mononuclear cells as part of the BeatAML cohort. Data in Fig. 3 and Supplementary Fig. 3 were generated as part of the BeatAML cohort and downloaded from their online browser (http://vizome.org/aml2). Data points were replotted using GraphPad Prism v10.

### Statistical analysis

Wilcoxon rank tests were used to assess significant differences in scDNA +Protein protein expression (Fig. 2, Supplementary Fig. S1). Survival data were analyzed by logrank (Mantel–Cox) tests (Fig. 3, Supplementary Fig. S2). Comparisons of area under the curve (AUC) metrics from the BeatAML ex vivo drug data were analyzed by Kruskal–Wallis tests (Fig. 3D, E, Supplementary Fig. S3) or Mann–Whitney tests (Fig. 3H, I) when appropriate.

### Plotting and graphical representations

**Fig. 1:**
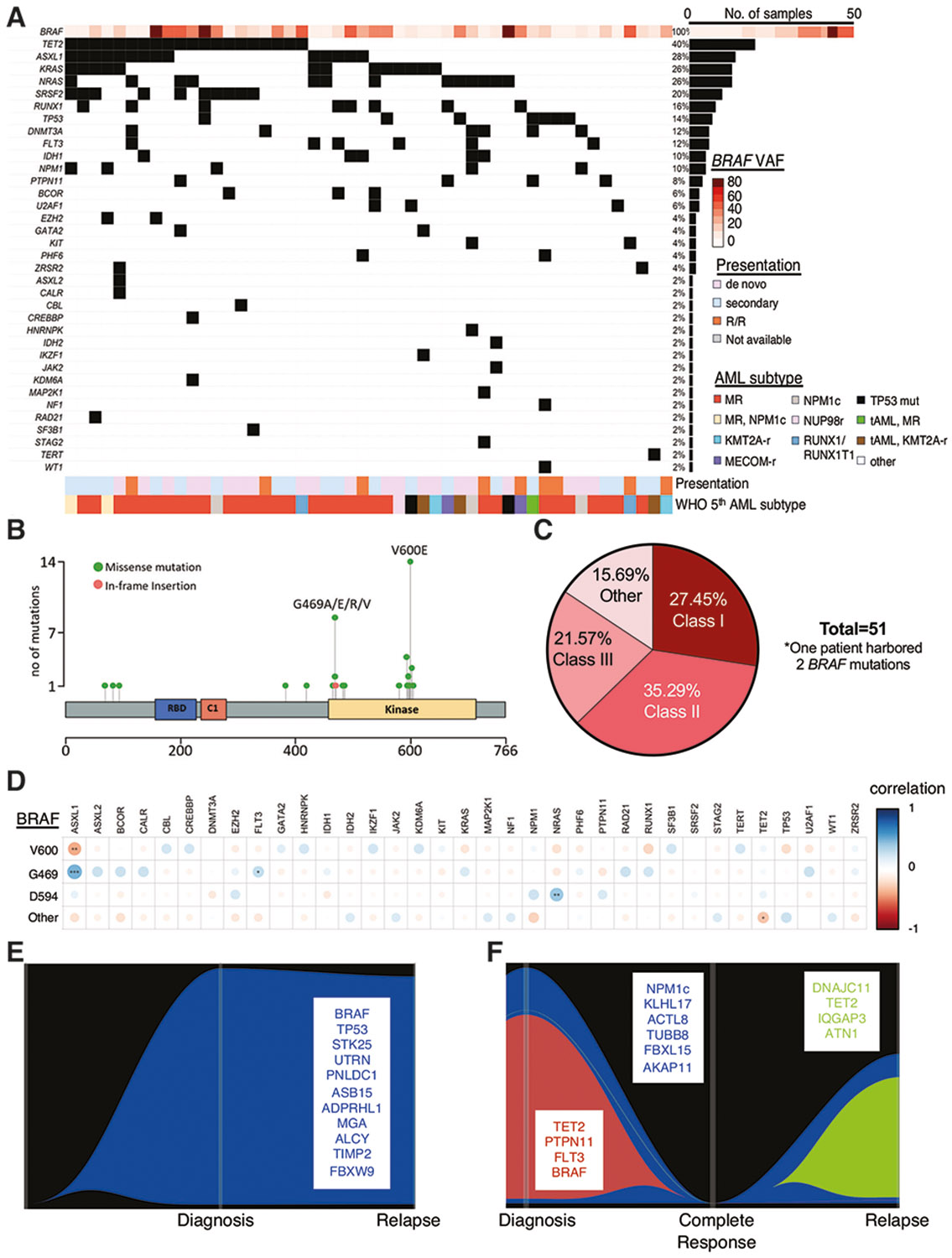
Non-canonical *BRAF* mutations in AML. **A** Oncoprint of 50 *BRAF*-mutant AML patients and co-occurring mutations ranked by frequency in cohort (co-mutations present in ≥5% of cohort shown for clarity). AML type and disease presentation depicted on lower panel. **B** Lollipop plot depicting frequency, gene location, and amino acid change of *BRAF* mutations identified in *BRAF*-mutant AML cohort. **C** Pie graph depicting frequencies of canonical class I (V600E), non-canonical class II (including G469), and non-canonical class III (including D594) mutations. Mutations with no prior class distinction are denoted as other. **D** Dot plot of enrichment (blue) or mutual exclusivity (brown) of AML co-mutations in each *BRAF* mutation class. Circles are colored and sized according to Pearson’s correlation coefficient, with *P*-values denoted as *P* < 0.05, *; *P* < 0.01, **; *P* < 0.001, ***. **E, F** Fish plots of mutations identified from whole-exome sequencing of two *BRAF*-mutant AML patients for whom paired relapse samples were available. Non-synonymous mutations identified are listed for each timepoint denoted by corresponding colors to identified clones.

Oncoprint (Fig. 1A) was generated using R oncoPrint package. Clonographs and UMAPs were generated in R. Graphs unless otherwise noted were generated using GraphPad Prism v10. UMAP data were plotted using the R ggplot2 package (RRID:SCR_014601). Other data processing was performed in R utilizing packages including: tidyr, dplyr, RColorbrewer, pals, and cowplot.

### Code availability & data sharing statement

**Fig. 2:**
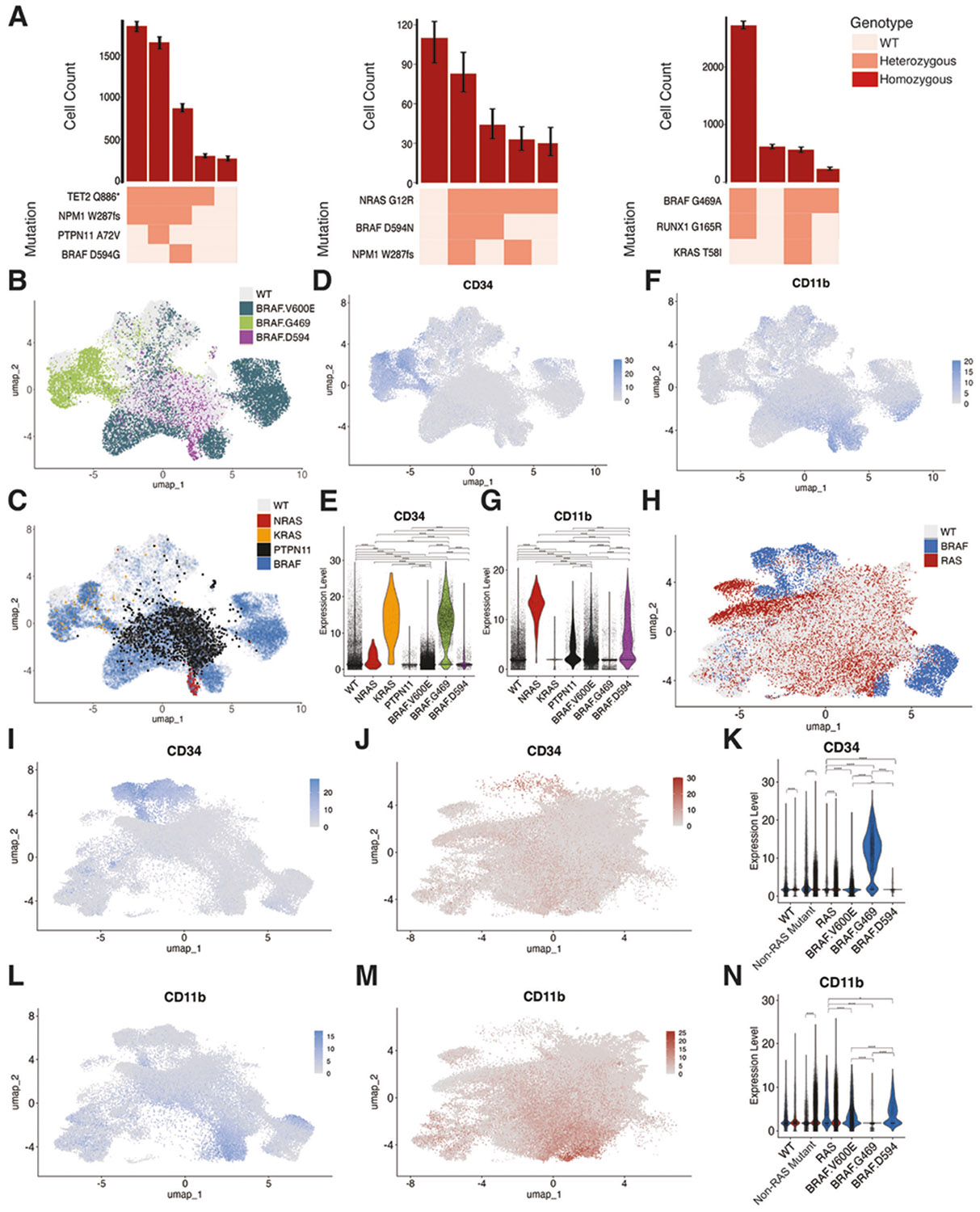
Genotype-immunophenotype relationships in *BRAF*-mutant AML clones. **A** Clonographs from 3 representative *BRAF*-mutant AML patients. Clones were quantified and denoted in columns (upper bar plot) with genotype of the clone depicted in bottom heatmap. Zygosity of mutation in heatmap denoted as wild-type (WT; beige), heterozygous (orange), or homozygous (red). **B, C** UMAP of cells from *BRAF*-mutant AML patient samples (*n* = 7) clustered by immunophenotype. Cells are denoted by (**B**) absence (gray) or presence of class I V600 (dark blue), class II G469 (green), or class III D594 (purple) *BRAF* mutations or (**C**) absence (gray) or presence of *NRAS* (red), *PTPN11* (black), *KRAS* (yellow), or *BRAF* (blue) mutations. UMAPs (**D, F**) and corresponding violin plots (**E, G**) of cells from A denoting low (gray) or high (purple) expression of CD34 (**D, F**) or CD11b (**E, G**) with colors in (**F**) and (**G**) denoted by genotype: WT (gray), *NRAS* (red), *PTPN11* (black), *KRAS* (yellow), *BRAF V600* (dark blue), *BRAF G469* (green), and *BRAF D594* (purple). Each dot within violin plot represents a cell. **H** UMAPs of cells from A (*n* = 7 samples) and *BRAF*-WT/RAS-mutant cells (*n* = 6) clustered by immunophenotype. Cells are denoted by absence (gray) or presence of *BRAF* (blue) or other RAS pathway (red) mutations. Faceted UMAP from (**H**) denoting *BRAF*-mutant cells (**l**) and *BRAF*-WT/RAS-mutant cells (**J**) with low (gray) or high (red) expression of CD34. K Corresponding violin plot for CD34 expression from cells in (**I, J**). Faceted UMAP from (**H**) denoting *BRAF*-mutant cells (**L**) and *BRAF*-WT/RAS-mutant cells (**M**) with low (gray) or high (red) expression of CD11b. **N** Corresponding violin plot for CD11b expression from cells in (**L, M**). Asterisks denote *P* < 0.05, *; *P* < 0.01, **; *P* < 0.001, ***, *P* < 0.0001, **** (**E, G, K, N**).

All scripts are available at https://github.com/bowmanr/scDNA and processed data files are available at https://github.com/plask3189/scProtein_Analysis for DNA+Protein analyses. Raw data files are available upon request from the authors. Data files from *BRAF WT* AML samples used for comparisons in Fig. 2 are available on dbGAP (phs004401.v1p1).

## RESULTS

### Clinical analysis shows enrichment of *BRAF* mutations in AML-MR

We identified 50 patients with *BRAF* mutations (1% of all patients with AML treated at our centers during the study period) including 21 (42%) with newly diagnosed AML, 9 (18%) with relapsed/refractory AML, and 20 (40%) with newly diagnosed secondary AML, defined as having a documented antecedent chronic myeloid neoplasm or therapy-related AML. *BRAF*-mutant AML patients had a median age of 67 years (range, 19–84 years), and 54% of patients were male (Supplementary Table 1). The median variant allele frequency for all *BRAF* mutants was 15% (range, 1–83%). The median bone marrow blast percentage was 43% (range, 3–93%), and the median absolute monocyte count was 0.52/μL (range, 0–94.56). *BRAF* mutations were enriched in patients with AML-MR based on both the World Health Organization (WHO 5th) [13] (*n* = 34/50, 68%) and the International Consensus Classification (ICC) [14] (*n* = 26/50, 52%) classifications, including 11 (22%) patients who had a history of a previous MDS/MPN.

### Molecular profiling of *BRAF*-mutant AML patients

The most frequent co-mutations in *BRAF*-mutant AML patients were in *TET2* (45%) and *ASXL1* (30%) (Fig. 1A). Oncogenic RAS pathway mutations were also common, with *NRAS* detected in 30% and *KRAS* in 23% of patients. In fact, 50% of *BRAF*-mutant patients (*n* = 25/50) harbored at least one other oncogenic RAS/MAPK pathway mutation, suggesting RAS pathway addiction and that the RAS pathway in general may be a convergent mechanism of leukemogenesis for this AML subtype.

Surprisingly, the majority (37/50, 73%) of *BRAF*-mutant cases were non-V600E variants (Fig. 1B, C). We found that 35 and 22% of patients harbored class II (*n* = 18) and class III (*n* = 11) mutations, respectively, with G469 (class II) and D594 (class III) mutations most common. This is in contrast to prior findings in solid tumors and lymphoid malignancies, where the V600E mutation represented the majority of *BRAF* alterations [4]. As class II and class III mutations have different dependencies on RAS-pathway activation, we next examined co-mutations across the *BRAF* mutation classes identified in our cohort (Fig. 1D). We found that indeed patients with class III D594 mutations were significantly enriched with *NRAS* co-mutations (*P* = 0.003). In contrast, class I V600 and class II G469 mutations seldom co-occurred with other RAS pathway mutations. Additionally, while *BRAF* V600 mutations were mutually exclusive with *ASXL1* co-mutations (*P* = 0.005), class II G469 mutations were significantly enriched in *ASXL1* co-mutated cases (*P* = 0.0003). The *BRAF* class-specific co-mutational patterns with common myeloid malignancy mutations are suggestive of unique pathogenesis compared to other AML subtypes.

### Whole-exome sequencing uncovers recurrent RAS-pathway addiction in *BRAF*-mutant AML

We performed whole-exome sequencing (WES) in a subset of patients (*n* = 5) to identify mutations outside of the targeted sequencing panels. *BRAF*-mutant AML harbored a median mutational burden of 22 non-synonymous, coding mutations (range 13–32). Interestingly, patients on average harbored 3 *RAS/MAPK* pathway mutations (range, 2–4; Supplementary Table 2). In two patients, WES was also performed at relapse. In one patient, the *BRAF* mutation was stable at relapse (Fig. 1E). In the second patient, the *BRAF* mutation was lost at relapse, but the patient instead acquired an *IQGAP3* mutation (Fig. 1F). IQGAP3 regulates cell proliferation by modulating the RAS/ERK signaling pathway, acting downstream of Rho family GTPases and interacting with ERK to promote cell cycle progression [25]. These findings support a convergent reliance on the MAPK/RAS pathway in this leukemia subset.

### Single-cell molecular profiling uncovers unique co-mutation and immunophenotypic patterns

To resolve the clonal architecture of *BRAF*-mutant AML, we performed simultaneous single-cell DNA (scDNA) + cell surface protein analysis on 7 samples using the Mission Bio Tapestri platform [21, 26]. We found that *BRAF* mutations could be identified either in the dominant, largest non-wild-type clone (*n* = 4) or in a sub-clone (*n* = 3) (Fig. 2A). *BRAF* mutations co-occurred on a single-cell level with oncogenic *NRAS* and *KRAS* mutations but were mutually exclusive with a *PTPN11* mutation in one sample. This observation is significantly divergent from the clonal co-mutation patterns we previously observed in AML patients harboring signaling mutations (*RAS, FLT3*), where we very rarely observed more than one signaling mutation in a clone [21].

We next investigated immunophenotype patterns in *BRAF*-mutant AML samples. We found that *BRAF* mutations were present in cells spanning different lineage states, highly dependent on the type of *BRAF* mutation (Fig. 2B). Similarly, we found that other RAS pathway co-mutations in *BRAF*-mutant samples spanned different lineage states (Fig. 2C). Specifically, we found *BRAF G469* mutant clones to be significantly enriched for a stem/progenitor phenotype with CD34 expression (*P* < 2 × 10^−16^) (Fig. 2D, E). KRAS-mutant cells were also significantly enriched in expressing CD34 in *BRAF*-mutant samples (*P* < 2 × 10^−16^). Conversely, *BRAF V600E* and *BRAF D594* mutant clones were enriched in monocytic cells expressing CD16 and/or CD11b (*P* <2×10^−16^ for all) (Fig. 2F, G, Supplementary Fig. S1A). *NRAS* and *PTPN11* mutant clones were also found to have increased CD11b and CD16 expression, as previously identified [21, 26, 27] (Fig. 2E-G, Supplementary Fig. S1A, B). Compared to BRAF WT clones, all *BRAF*-mutant clones had higher CD123 expression (Supplementary Fig. S1C, D). *BRAF V600E* mutant clones, specifically, displayed higher expression of FLT3 compared to class II and class III mutant clones (Supplementary Fig. S1E, F). These findings suggest that *BRAF* mutations from different classes may lead to highly divergent cell states and immunophenotypes of the resulting AMLs.

We then compared our *BRAF*-mutant AML samples to a separate subset of *BRAF WT/RAS*-mutant AML samples previously analyzed by scDNA+Protein [23], which harbor similar AML co-mutations found in a subset of our *BRAF*-mutant AML patients, including *NRAS* mutations (Fig. 2H). We found that *BRAF G469* mutant cells had overall higher expression of CD34 compared with *NRAS*-mutant cells from both sample cohorts (*P* < 2 × 10^−16^; Fig. 2I-K, Supplementary Fig. S1G). Interestingly, even though *BRAF V600* and *BRAF D594* mutant clones expressed CD11b and CD14, the *NRAS*-mutant cells from *BRAF* WT/RAS-mutant samples expressed significantly higher levels of the monocyte markers (*NRAS* vs *BRAF V600, P* < 2 × 10^−16^; *NRAS* vs *BRAF D594 P* = 0.039) (Fig. 2L-N, Supplementary Fig. S1G-J). *BRAF V600* mutant clones had expression of CD123 comparable to that of RAS mutant clones from *BRAF WT/RAS*-mutant AML samples (Supplementary Fig. S1G, K-M).

### *BRAF* mutations are associated with poor overall survival regardless of treatment regimen

**Fig. 3:**
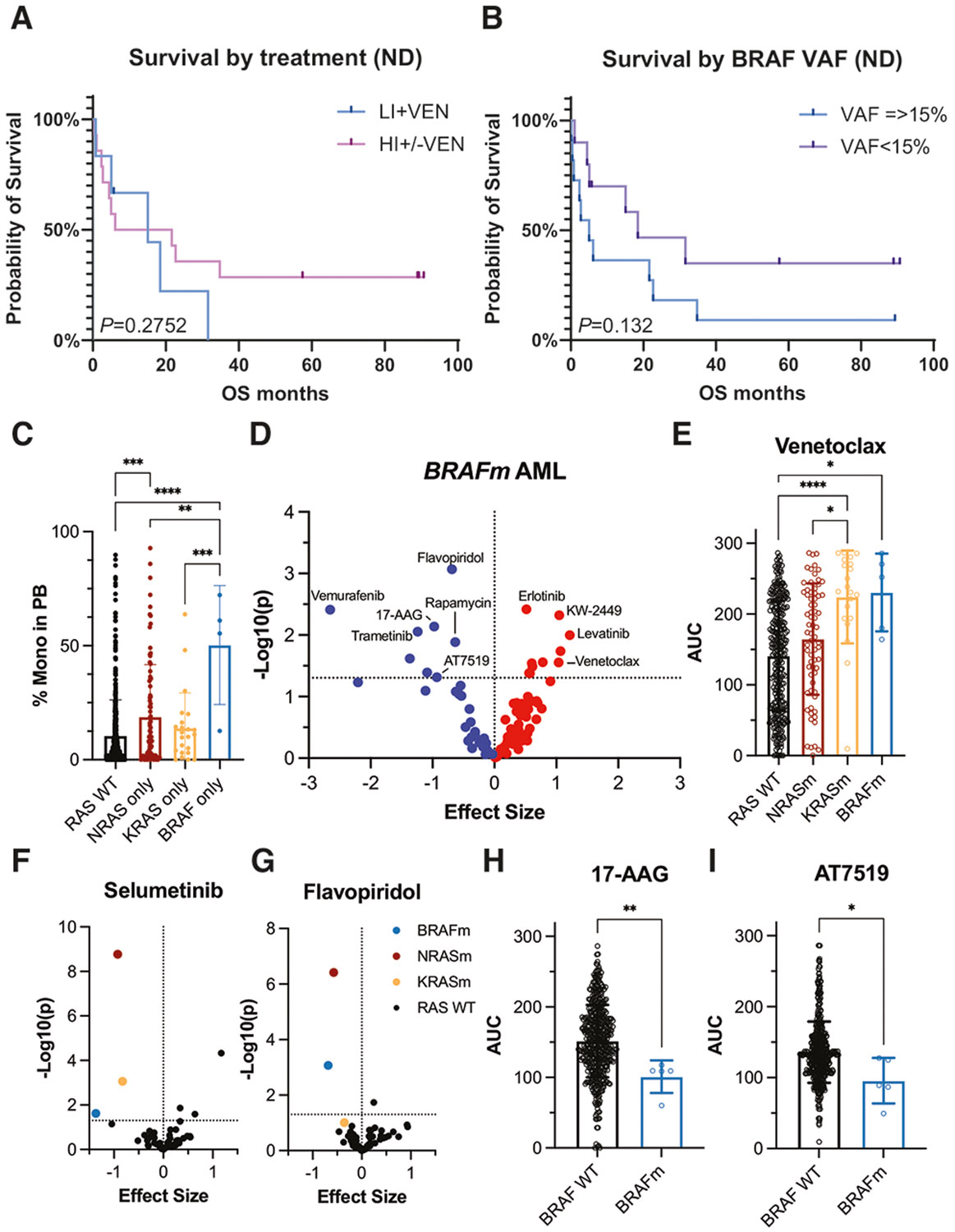
*BRAF*-mutant AML patients have significantly poor overall survival and unique drug sensitivities. **A** Kaplan–Meier survival curve of *BRAF*-mutant patients treated with low-intensity chemo +/− venetoclax (LI+/−VEN; blue) or high-intensity chemo +/− venetoclax (HI + VEN; pink). *P*-value calculated by logrank test. **B** Kaplan–Meier survival curve of ND *BRAF*-mutant patients stratified by *BRAF* mutant variant allele frequency (VAF). Patients with a VAF greater than or equal to 15 (*n* = 25, blue) are compared to patients with a VAF less than 15 (*n* = 25, purple). *P*-value calculated by logrank test. **C** Violin plot showing percentages of monocytes in the peripheral blood for BeatAML cohort newly diagnosed AML patients harboring *BRAF* (*n* = 3; blue), *NRAS* (*n* = 64; red), and *KRAS* (*n* = 22; yellow) mutations compared to *RAS-WT* AML patients (*n* = 433; black). **D** Volcano plot showing increased sensitivity (blue) or resistance (red) of primary AML samples harboring *BRAF* mutations (both V600E and non-canonical mutations) to short term ex vivo drug treatments by plotting Glass’ Delta effect size compared to all other tested AML samples (BRAF-WT). Dotted line indicates *P* value = 0.05. Data from BeatAML cohort. Specific drugs discussed in the text are highlighted. **E** Bar plot denoting area under the curve (AUC) results for *RAS WT* (black, *n* = 295), *NRAS*-mutant (red; *n* = 69), KRAS-mutant (yellow; *n* = 21), or *BRAF*-mutant (blue; *n* = 5) samples treated with venetoclax. Volcano plots of the relative sensitivity/resistance of *BRAF* (blue), *NRAS* (red), and *KRAS* (yellow) mutant AML samples to Selumetinib (**F**), and Flavopiridol (**G**) compared to samples from all other *RAS WT* AML genotypes (black). Dotted line indicates *P* value = 0.05. Bar plots depicting AUC results for *BRAF*-WT (black) and *BRAF*-mutant (blue) samples treated with 17-AAG (**H**) or AT7519 (**I**). Asterisks denote *P* < 0.05, *; *P* < 0.01, **; *P* < 0.001, ***, *P* < 0.0001, **** (**D, F, H, I**).

The overall response rate (CR, CRi, or CRh) was 46% and the median OS was 5.67 months (range, 0.13–90.7 months) for the entire cohort (Supplementary Table 3, Supplementary Fig. S2A). Newly diagnosed (ND) *BRAF*-mutant patients were treated with a variety of low-intensity (LI, *n* = 7) and high-intensity (HI, *n* = 14) treatment regimens with or without venetoclax [28] (Fig. 3A). ND *BRAF*-mutant AML patients had poor overall survival (OS) regardless of therapeutic regimen (*P* = 0.2752; LI+/−VEN: 15.09 months, 95% Cl 0.13-non-estimable (NE) vs HI+/−VEN: 13.90 months, 95% Cl 2.37-NE). *BRAF*-mutated patients with de novo AML treated with HI regimens had OS similar to that of 2022 European LeukemiaNet [29] (ELN) Intermediate-risk patients (Supplementary Fig. S2B). *BRAF* variant allele burden did not have an impact on OS in newly diagnosed patients (VAF ≥15%: 5.02 months, 95% CI: 0.43–22.68 vs VAF < 15%: 18.47 months, 95% CI: 1.02 to NE; *P* = 0.132; Fig. 3B). Prior exposure to venetoclax among R/R AML did not impact OS (Supplementary Fig. S2C, *P* = 0.884). We next reviewed the BeatAML cohort [2, 15] and identified three ND patients with *BRAF* mutations. Their OS was very short, with all three patients deceased at 4 months post-study enrollment. Interestingly, patients with *BRAF*-mutant AML from the BeatAML cohort had significantly higher percentages of monocytes (*P* < 0.0018) in the peripheral blood compared to *NRAS/KRAS*-mutant patients (Fig. 3C).

### Ex vivo drug sensitivity data suggest *BRAF*-specific vulnerabilities

To further evaluate treatment options in *BRAF*-mutant AML, we looked at relative drug efficacies using the samples in the BeatAML cohort [2, 15]. Five *BRAF*-mutant AML samples were tested, including 3 harboring V600E mutations and 2 harboring class II G469 mutations (Fig. 3D). *BRAF*-mutant AML samples were significantly more resistant to 8 therapeutic agents compared with *BRAF*-WT AML samples. These drugs included Erlotinib, an EGFR inhibitor, and two multi-kinase inhibitors, KW-2449 and Lenvatinib. Interestingly, we also found that *BRAF*-mutant AML samples showed increased resistance to venetoclax compared to *RASWT* patients, providing a rationale for the poor responses to venetoclax-based regimens seen in our patients (*P* = 0.049; Fig. 3A, E, Supplementary Fig. S3A). *NRAS/KRAS*-mutant AMLs have been previously shown to be resistant to venetoclax-based regimens and showed resistance levels similar to *BRAF*-mutant AML [27, 30-32]. Conversely, these samples were found to have significantly increased sensitivity to 8 therapeutic agents compared to *BRAFWT* AML, with the highest sensitivity to flavopiridol, a cyclin-dependent kinase (CDK) inhibitor [33], which had previously shown exciting efficacy data in specific AML subtypes, and had been more recently suggested as a strategy to overcome venetoclax resistance [34]. Not surprisingly, additional efficacies were observed upon treatment with vemurafenib, a mutant BRAF V600E inhibitor [35], trametinib, a MEK inhibitor [36], and rapamycin, an mTOR complex inhibitor [37], and also with 17-AAG, an HSP90 inhibitor [38].

We compared the drug efficacies of *BRAF*-mutant AML to those identified in *NRAS/KRAS*-mutant AML samples. *RAS*- and *BRAF*-mutant samples all shared sensitivities to Trametinib, Selumetinib, and CI-1040, compared to other AML genotypes (Fig. 3F, Supplementary Fig. S3B). *NRAS*- and *BRAF*-mutant AML shared sensitivity to flavopiridol, but *KRAS*-mutant AMLs were not significantly sensitive (Fig. 3G). Conversely, *NRAS*-mutant AML was sensitive to panobinostat; however, the responses of *KRAS*- and *BRAF*-mutant AML did not reach statistical significance (Supplementary Fig. S3C). *BRAF*-mutant AML showed unique sensitivity to the HSP90 inhibitor, 17-AAG and the broad CDK inhibitor, AT7519, compared to *BRAF WT* AMLs and trended towards significance with *RAS*-mutant AMLs (Fig. 3H, I, Supplementary Fig. S3D). These findings suggest that *BRAF*-mutant AML may have unique drug sensitivities that extend beyond BRAF/RAF pathway inhibition, with specific promise of both HSP90 and broad CDK inhibition that should be studied further as alternative therapeutic options for patients.

## DISCUSSION

Oncogenic mutations in the RAS/MAPK cascade occur in almost all types of human cancer. Alterations in the *BRAF* gene occur in ~15% of all human cancers [4]. Interestingly, while RAS pathway mutations are frequent in AML occurring in ~20% of cases, mutations in the gene encoding the downstream effector kinase *BRAF* occur much less frequently [1, 3]. This is in contrast to many solid malignancies where *BRAF* mutations represent significant cancer drivers, and to the B-cell derived hairy cell leukemia, where *BRAF V600* mutations occur in almost 100% of cases. However, almost 75% of the *BRAF* mutations in our patient cohort were non-canonical mutations that were not located at the V600 hotspot. Since *BRAF V600E* mutation makes up 70–80% of all *BRAF* gene alterations in human cancer, our findings suggest that AML is unique in being enriched for non-hotspot *BRAF* mutations.

BRAF mutant proteins have been classified based on the mutation location and the intrinsic kinase activity of the mutated protein. Class I (V600) and class II (i.e. G469 and others) mutants are hyperactive and RAS-independent [8, 9], whereas class III (i.e. D594 and others) mutants are inactive or kinase-dead and require an activated or oncogenic RAS protein [11, 12]. Indeed, *BRAF D594* mutant AML patients in our cohort frequently harbored *NRAS* co-mutations, and our single-cell data from a *BRAF D594* mutant patient showed that the *BRAF* and *NRAS* mutations co-occurred in the same clone, indicating the potential dependence of *BRAF D594* mutations on activated RAS in AML as well. Interestingly, we observed divergent co-mutation patterns between V600 and G469 mutations, particularly with *ASXL1* co-mutations, suggesting the *BRAF* mutation classes may divergently synergize with recurrent AML mutations. Similarly, we also found unique immunophenotype patterns between the different *BRAF* mutant clones, with *BRAF G469* mutant clones enriched in CD34 expression and *BRAF D594* mutant clones having higher expression of CD11b and CD16. Additionally, our *BRAF*-mutant AML samples showed higher levels of CD123 expression, which warrants testing the efficacy of recent CD123-targeted therapies [39]. Not only do these findings suggest that the *BRAF* mutations may be prevalent with certain co-mutations based on synergistic interactions, but also that *BRAF* mutant classes are enriched in distinct leukemic cell compartments. Although limited by the number of samples available, the results of our multiomic analysis underscore the divergent phenotypes among AML samples with different *BRAF* mutations and suggest that further studies are needed to determine how the *BRAF* mutations interact with recurrent AML drivers and affect the phenotype of the leukemia.

While *RAS* mutations in other cancers typically bestow poor patient outcomes, the prognostic relevance of *RAS* mutations remains less clear in AML. *NRAS*-mutant AML patients typically show superior responses to standard chemotherapy compared with patients with other signaling mutations [3], but *RAS/MAPK* pathway mutations are also enriched in AML patients who are either refractory to or who develop resistance to targeted and novel therapies. *BRAF* mutations in AML have been associated with poor prognoses [40-42]; however, many studies lacked sufficient numbers of *BRAF*-mutated cases for analysis. Although our study still includes a relatively small number of patients, which precludes us from performing a multivariable outcome analysis, it also shows that *BRAF*-mutated AML patients have poor outcomes of *BRAF*-mutated AML patients, regardless of the frontline regimen, which is consistent with the literature [3, 40-42]. These findings align with the recurrent acquired resistance/relapse mechanism for the venetoclax/azacitidine combination treatment, which lies in the expansion of a myelomonocytic blast population enriched for *NRAS/MAPK* mutations [27, 31]. This is further supported by our drug sensitivity data on *BRAF*-mutant AML obtained from the BeatAML cohort, which suggests that cells harboring *BRAF* mutations are more resistant to venetoclax; however, it is still unknown if they utilize similar mechanisms as *N/KRAS*-mutant cells. These findings suggest that *BRAF* mutations, like other *RAS/MAPK* mutations, appear to play a significant role in therapy resistance and may serve as a convergent mechanism for therapy escape in AML.

Studies in other cancer subtypes have uncovered that each class of *BRAF* mutant proteins has distinct biochemical and signaling properties, which may lead to different dependencies on interacting proteins and alternative pathways. For example, these studies suggest that class III BRAF mutants are sensitive to RAS-pathway inhibition [12], whereas the class II BRAF mutants may be sensitive to MAPK-pathway inhibitors outside of RAS-targeted compounds [43]. Data from the BeatAML study uncovered multiple therapeutic agents that may hold promise in *BRAF*-mutant AML patients, including HSP90 and CDK inhibition. However, the majority of the 5 samples harbored the V600E mutation, and no samples tested contained a class III mutation. Therefore, further examination of these agents in class II and III mutant AML samples is required to fully characterize their efficacy as potential avenues for *BRAF*-mutant AML treatment.

In conclusion, our study demonstrates that while rare, *BRAF* mutations are recurrent in AML, and are typically non-canonical. They exhibit distinct, class-specific co-mutation and immunophenotypic patterns, and are associated with poor survival outcomes, even in newly diagnosed patients. Ex vivo drug sensitivity studies identified potential therapeutic agents for *BRAF*-mutant AML. These findings highlight the unique nature of *BRAF*-mutated AML, underscoring the critical need for improved understanding of this genotype to enhance survival outcomes, and the inclusion of expanded *BRAF* gene coverage into routine testing panels for newly diagnosed and relapsed/refractory AML patients.

## Supplementary Material

Supplemental Figure 1

Supplementary Figures & Tables

Supplementary Table 3

Supplemental Figure 2

Supplemental Figure 3

**Supplementary information** The online version contains supplementary material available at https://doi.org/10.1038/s41375-026-02996-1.

## References

[1] Burgess MR, Hwang E, Firestone AJ, Huang T, Xu J, Zuber J, Preclinical efficacy of MEK inhibition in Nras-mutant AML. Blood. 2014;124:3947–55. 25361812 10.1182/blood-2014-05-574582 PMC4271180

[2] Tyner JW, Tognon CE, Bottomly D, Wilmot B, Kurtz SE, Savage SL, Functional genomic landscape of acute myeloid leukaemia. Nature. 2018;562:526–31. 30333627 10.1038/s41586-018-0623-z PMC6280667

[3] Papaemmanuil E, Gerstung M, Bullinger L, Gaidzik VI, Paschka P, Roberts ND, Genomic classification and prognosis in acute myeloid leukemia. N Engl J Med. 2016;374:2209–21. 27276561 10.1056/NEJMoa1516192 PMC4979995

[4] Turski ML, Vidwans SJ, Janku F, Garrido-Laguna I, Munoz J, Schwab R, Genomically driven tumors and actionability across histologies: BRAF-mutant cancers as a paradigm. Mol Cancer Ther. 2016;15:533–47. 27009213 10.1158/1535-7163.MCT-15-0643

[5] Dankner M, Rose AAN, Rajkumar S, Siegel PM, Watson IR. Classifying BRAF alterations in cancer: new rational therapeutic strategies for actionable mutations. Oncogene. 2018;37:3183–99. 29540830 10.1038/s41388-018-0171-x

[6] Hodis E, Watson IR, Kryukov GV, Arold ST, Imielinski M, Theurillat JP, A landscape of driver mutations in melanoma. Cell. 2012;150:251–63. 22817889 10.1016/j.cell.2012.06.024 PMC3600117

[7] Tiacci E, Trifonov V, Schiavoni G, Holmes A, Kern W, Martelli MP, BRAF mutations in hairy-cell leukemia. N Engl J Med. 2011;364:2305–15. 21663470 10.1056/NEJMoa1014209 PMC3689585

[8] Davies H, Bignell GR, Cox C, Stephens P, Edkins S, Clegg S, Mutations of the BRAF gene in human cancer. Nature. 2002;417:949–54. 12068308 10.1038/nature00766

[9] Ng PK, Li J, Jeong KJ, Shao S, Chen H, Tsang YH, Systematic functional annotation of somatic mutations in cancer. Cancer Cell. 2018;33:450–62.e10. 29533785 10.1016/j.ccell.2018.01.021 PMC5926201

[10] Lavoie H, Jin T, Lajoie D, Decossas M, Gendron P, Wang B, BRAF oncogenic mutants evade autoinhibition through a common mechanism. Science. 2025;388:eadp2742. 40440367 10.1126/science.adp2742

[11] Heidorn SJ, Milagre C, Whittaker S, Nourry A, Niculescu-Duvas I, Dhomen N, Kinase-dead BRAF and oncogenic RAS cooperate to drive tumor progression through CRAF. Cell. 2010;140:209–21. 20141835 10.1016/j.cell.2009.12.040 PMC2872605

[12] Yao Z, Yaeger R, Rodrik-Outmezguine VS, Tao A, Torres NM, Chang MT, Tumours with class 3 BRAF mutants are sensitive to the inhibition of activated RAS. Nature. 2017;548:234–8. 28783719 10.1038/nature23291 PMC5648058

[13] Khoury JD, Solary E, Abla O, Akkari Y, Alaggio R, Apperley JF, The 5th edition of the World Health Organization Classification of Haematolymphoid Tumours: Myeloid and Histiocytic/Dendritic Neoplasms. Leukemia. 2022;36:1703–19. 35732831 10.1038/s41375-022-01613-1 PMC9252913

[14] Arber DA, Orazi A, Hasserjian RP, Borowitz MJ, Calvo KR, Kvasnicka HM, International Consensus Classification of Myeloid Neoplasms and Acute Leukemias: integrating morphologic, clinical, and genomic data. Blood. 2022;140:1200–28. 35767897 10.1182/blood.2022015850 PMC9479031

[15] Bottomly D, Long N, Schultz AR, Kurtz SE, Tognon CE, Johnson K, Integrative analysis of drug response and clinical outcome in acute myeloid leukemia. Cancer Cell. 2022;40:850–64.e9. 35868306 10.1016/j.ccell.2022.07.002 PMC9378589

[16] Ok CY, Singh R, Luthra R, Hatfield D, Floyd K, Loghavi S, Endleukemia assay v1: enabling NGS-based comprehensive routine molecular profiling of leukemias in routine clinical care. Blood. 2017;130:2679.

[17] Montalban-Bravo G, Kanagal-Shamanna R, Benton CB, Class CA, Chien KS, Sasaki K, Genomic context and TP53 allele frequency define clinical outcomes in TP53-mutated myelodysplastic syndromes. Blood Adv. 2020;4:482–95. 32027746 10.1182/bloodadvances.2019001101 PMC7013259

[18] Eisfeld AK, Mrozek K, Kohlschmidt J, Nicolet D, Orwick S, Walker CJ, The mutational oncoprint of recurrent cytogenetic abnormalities in adult patients with de novo acute myeloid leukemia. Leukemia. 2017;31:2211–8. 28321123 10.1038/leu.2017.86 PMC5628133

[19] Stiff A, Fornerod M, Kain BN, Nicolet D, Kelly BJ, Miller KE, Multiomic profiling identifies predictors of survival in African American patients with acute myeloid leukemia. Nat Genet. 2024;56:2434–46. 39367245 10.1038/s41588-024-01929-x PMC11549055

[20] Qin G, Dai J, Chien S, Martins TJ, Loera B, Nguyen QH, Mutation patterns predict drug sensitivity in acute myeloid leukemia. Clin Cancer Res. 2024;30:2659–71. 38619278 10.1158/1078-0432.CCR-23-1674 PMC11176916

[21] Miles LA, Bowman RL, Merlinsky TR, Csete IS, Ooi AT, Durruthy-Durruthy R, Single-cell mutation analysis of clonal evolution in myeloid malignancies. Nature. 2020;587:477–82. 33116311 10.1038/s41586-020-2864-x PMC7677169

[22] Robinson TM, Bowman RL, Persaud S, Liu Y, Neigenfind R, Gao Q, Single-cell genotypic and phenotypic analysis of measurable residual disease in acute myeloid leukemia. Sci Adv. 2023;9:eadg0488. 37729414 10.1126/sciadv.adg0488 PMC10881057

[23] Drucker M, Lee D, Zhang X, Kain B, Bowman M, Nicolet D, Genotype-immunophenotype relationships in NPM1-mutant AML clonal evolution uncovered by single cell multiomic analysis. Blood 10.1182/blood.2025030772 (2026). PMC13386097 41950000

[24] Korsunsky I, Millard N, Fan J, Slowikowski K, Zhang F, Wei K, Fast, sensitive and accurate integration of single-cell data with Harmony. Nat Methods. 2019;16:1289–96. 31740819 10.1038/s41592-019-0619-0 PMC6884693

[25] Nojima H, Adachi M, Matsui T, Okawa K, Tsukita S, Tsukita S. IQGAP3 regulates cell proliferation through the Ras/ERK signalling cascade. Nat Cell Biol. 2008;10:971–8. 18604197 10.1038/ncb1757

[26] Morita K, Wang F, Jahn K, Hu T, Tanaka T, Sasaki Y, Clonal evolution of acute myeloid leukemia revealed by high-throughput single-cell genomics. Nat Commun. 2020;11:5327. 33087716 10.1038/s41467-020-19119-8 PMC7577981

[27] Sango J, Carcamo S, Sirenko M, Maiti A, Mansour H, Ulukaya G, RAS-mutant leukaemia stem cells drive clinical resistance to venetoclax. Nature. 2024;636:241–50. 39478230 10.1038/s41586-024-08137-x PMC11618090

[28] DiNardo CD, Pratz KW, Letai A, Jonas BA, Wei AH, Thirman M, Safety and preliminary efficacy of venetoclax with decitabine or azacitidine in elderly patients with previously untreated acute myeloid leukaemia: a non-randomised, open-label, phase 1b study. Lancet Oncol. 2018;19:216–28. 29339097 10.1016/S1470-2045(18)30010-X

[29] Dohner H, Wei AH, Appelbaum FR, Craddock C, DiNardo CD, Dombret H, Diagnosis and management of AML in adults: 2022 recommendations from an international expert panel on behalf of the ELN. Blood. 2022;140:1345–77. 35797463 10.1182/blood.2022016867

[30] Zhang Q, Riley-Gillis B, Han L, Jia Y, Lodi A, Zhang H, Activation of RAS/MAPK pathway confers MCL-1 mediated acquired resistance to BCL-2 inhibitor venetoclax in acute myeloid leukemia. Signal Transduct Target Ther. 2022;7:51. 35185150 10.1038/s41392-021-00870-3 PMC8858957

[31] Pei S, Pollyea DA, Gustafson A, Stevens BM, Minhajuddin M, Fu R, Monocytic subclones confer resistance to venetoclax-based therapy in patients with acute myeloid leukemia. Cancer Discov. 2020;10:536–51. 31974170 10.1158/2159-8290.CD-19-0710 PMC7124979

[32] Dohner H, Pratz KW, DiNardo CD, Wei AH, Jonas BA, Pullarkat VA, Genetic risk stratification and outcomes among treatment-naive patients with AML treated with venetoclax and azacitidine. Blood. 2024;144:2211–22. 39133921 10.1182/blood.2024024944 PMC11600046

[33] Karp JE, Passaniti A, Gojo I, Kaufmann S, Bible K, Garimella TS, Phase I and pharmacokinetic study of flavopiridol followed by 1-beta-D-arabinofuranosylcytosine and mitoxantrone in relapsed and refractory adult acute leukemias. Clin Cancer Res. 2005;11:8403–12. 16322302 10.1158/1078-0432.CCR-05-1201

[34] Stewart ML, Gibbs J, Watanabe-Smith K, Nguyen A, Kenna I, Thiel-Klare K, CDK4/6 inhibition overcomes venetoclax resistance mechanisms with enhanced combination activity in acute myeloid leukemia. Cell Rep Med. 2026;7:102526. 41468895 10.1016/j.xcrm.2025.102526 PMC12866115

[35] Bollag G, Hirth P, Tsai J, Zhang J, Ibrahim PN, Cho H, Clinical efficacy of a RAF inhibitor needs broad target blockade in BRAF-mutant melanoma. Nature. 2010;467:596–9. 20823850 10.1038/nature09454 PMC2948082

[36] Flaherty KT, Infante JR, Daud A, Gonzalez R, Kefford RF, Sosman J, Combined BRAF and MEK inhibition in melanoma with BRAF V600 mutations. N Engl J Med. 2012;367:1694–703. 23020132 10.1056/NEJMoa1210093 PMC3549295

[37] Guba M, von Breitenbuch P, Steinbauer M, Koehl G, Flegel S, Hornung M, Rapamycin inhibits primary and metastatic tumor growth by antiangiogenesis: involvement of vascular endothelial growth factor. Nat Med. 2002;8:128–35. 11821896 10.1038/nm0202-128

[38] Solit DB, Ivy SP, Kopil C, Sikorski R, Morris MJ, Slovin SF, Phase I trial of 17-allylamino-17-demethoxygeldanamycin in patients with advanced cancer. Clin Cancer Res. 2007;13:1775–82. 17363532 10.1158/1078-0432.CCR-06-1863 PMC3203693

[39] Daver NG, Montesinos P, DeAngelo DJ, Wang ES, Papadantonakis N, Todisco E, Pivekimab sunirine (IMGN632), a novel CD123-targeting antibody-drug conjugate, in relapsed or refractory acute myeloid leukaemia: a phase 1/2 study. Lancet Oncol. 2024;25:388–99. 38423051 10.1016/S1470-2045(23)00674-5 PMC11103591

[40] Abuasab T, Mohamed S, Pemmaraju N, Kadia TM, Daver N, DiNardo CD, BRAF mutation in myeloid neoplasm: incidences and clinical outcomes. Leuk Lymphoma. 2024;65:1344–9. 38696743 10.1080/10428194.2024.2347539

[41] George GV, Evans AG, Jajosky AN. BRAF V600E-mutant acute myeloid leukemia: a case series and literature review of a rare entity. Genes. 2024;15:1383. 39596583 10.3390/genes15111383 PMC11593820

[42] Fei F, Caporale C, Chang L, Fortini BK, Ali H, Bell D, BRAF mutations in patients with myeloid neoplasms: a cancer center multigene next-generation sequencing analysis experience. Int J Mol Sci. 2024;25:5183. 38791222 10.3390/ijms25105183 PMC11121641

[43] Dankner M, Lajoie M, Moldoveanu D, Nguyen TT, Savage P, Rajkumar S, Dual MAPK inhibition is an effective therapeutic strategy for a subset of class II BRAF mutant melanomas. Clin Cancer Res. 2018;24:6483–94. 29903896 10.1158/1078-0432.CCR-17-3384

