# Supplementary figures and images for: Prognostic and therapeutic implications of BRAF mutations in acute myeloid leukemia
Source: Leukemia. Author manuscript; Available in PMC 2026 Jul 31. (PMC13421326; doi:10.1038/s41375-026-02996-1)

Supplemental Figure 1

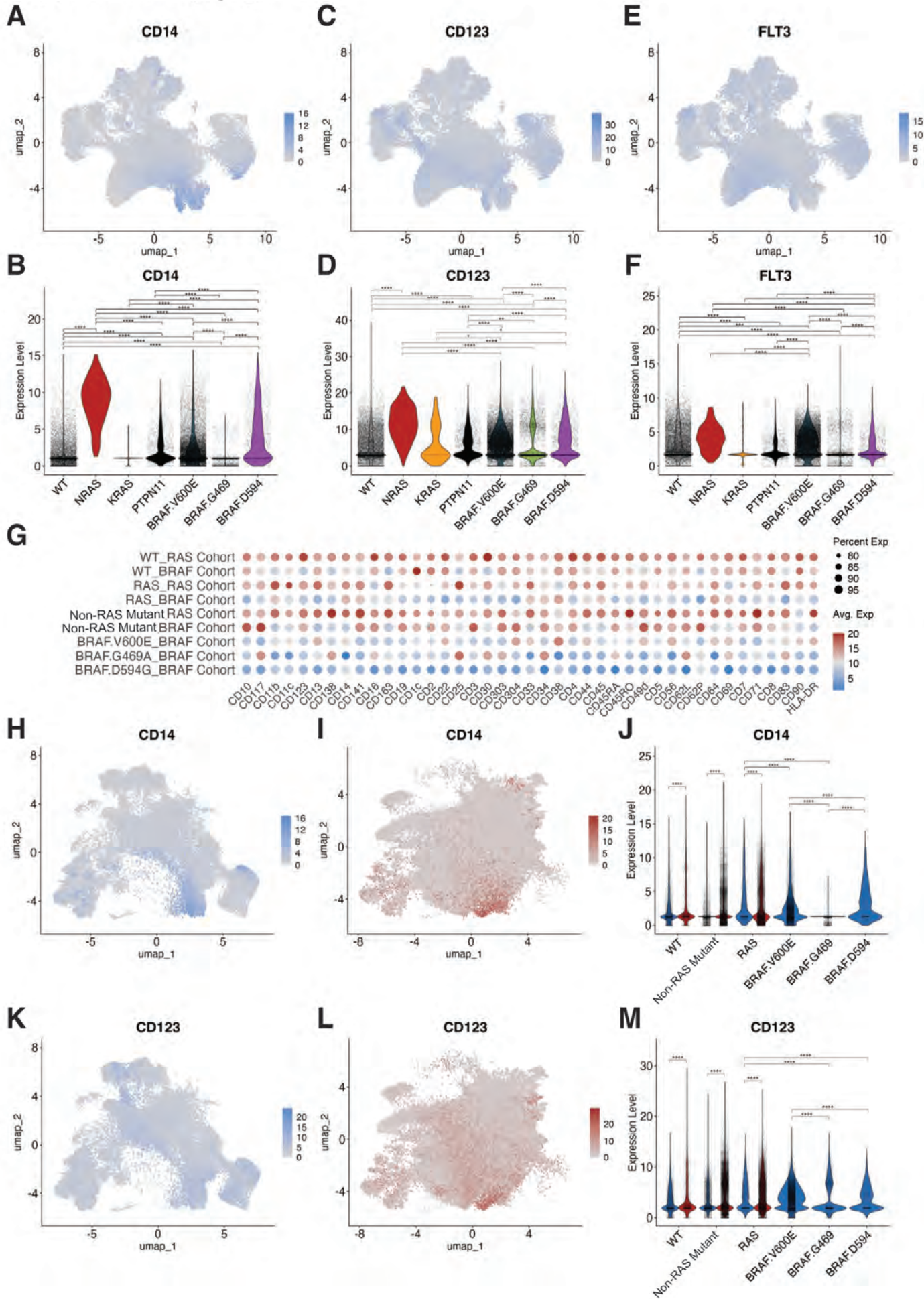

Supplement: Supplemental Figure 1 [file NIHMS2196509-supplement-Supplemental_Figure_1.pdf]

# Supplemental Figure 2

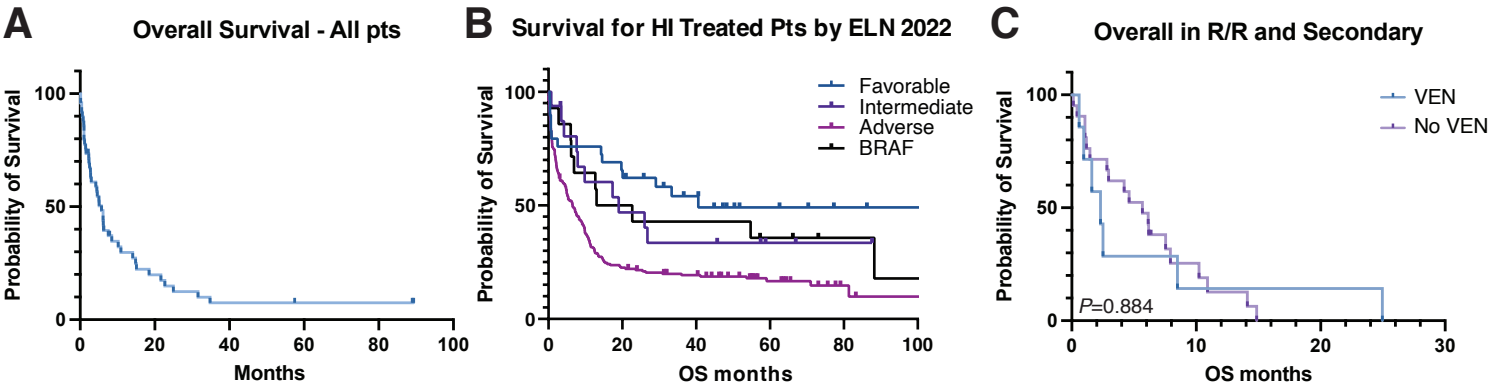

Supplement: Supplemental Figure 2 [file NIHMS2196509-supplement-Supplemental_Figure_2.pdf]

Supplemental Figure 3

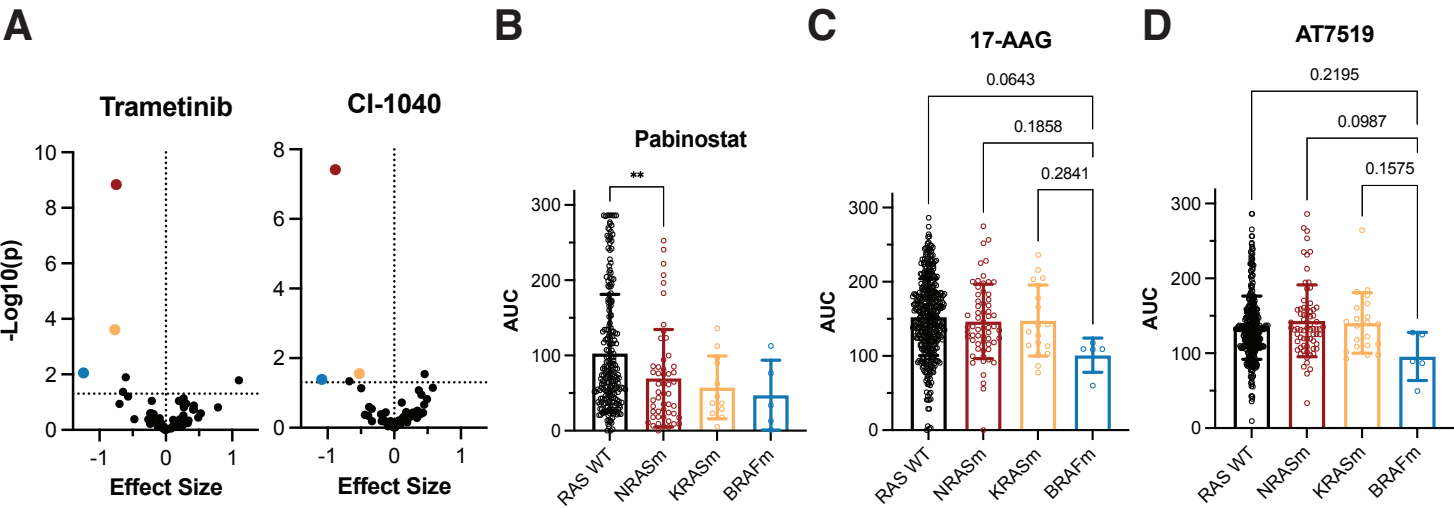

Supplement: Supplemental Figure 3 [file NIHMS2196509-supplement-Supplemental_Figure_3.pdf]
