# Supplementary material for: Prognostic and therapeutic implications of BRAF mutations in acute myeloid leukemia
Source: Leukemia. Author manuscript; Available in PMC 2026 Jul 31. (PMC13421326; doi:10.1038/s41375-026-02996-1)
Supplement: Supplementary Figures & Tables [file NIHMS2196509-supplement-Supplementary_Figures___Tables.docx]

**Supplementary Materials**

**Supplementary Figures & Tables**

**Supplementary Table 1.** Summary of pretreatment characteristics of *BRAF*-mutated patients with AML in our cohort.^*^

| **Demographic/Clinical Parameter** | ***BRAF*-mutant cohort** |
| --- | --- |
| Age at original diagnosis, years, median (range) | 67 (19-84) |
| <65 (%) | 23 (46) |
| ≥65 (%) | 27 (54) |
| Sex, number (%) |  |
| Female | 23 (46) |
| Male | 27 (54) |
| Disease at presentation, number (%) |  |
| De novo, newly diagnosed | 21 (42) |
| Relapse/refractory | 9 (18) |
| Secondary^†^ | 20 (40) |
| WHO Classification, number (%) |  |
| AML-MR | 34 (68) |
| AML with *NPM1* mutation | 3 (6) |
| AML with *MECOM*-rearrangement | 2 (4) |
| AML with *RUNX1:RUNX1T1* fusion | 2 (4) |
| t-AML with *KMT2A*-rearrangement | 3 (6) |
| Other | 6 (12) |
| ICC Classification, number (%) |  |
| AML-MR | 26 (52) |
| AML with mutated *NPM1* | 5 (10) |
| AML with mutated *TP53* | 6 (12) |
| AML-*KMT2A* | 5 (10) |
| AML with *MECOM*-rearrangements | 2 (4) |
| AML with *RUNX1:RUNX1T1* fusion | 2 (4) |
| Other | 4 (8) |
| Bone marrow blast percentage, % median (range) | 43 (3-93) |
| Absolute monocyte count, median (range) | 0.52 (0-94.56) |
| *BRAF* VAF, median (range) | 15 (1-83) |
| <15 (%) | 25 (50) |
| ≥15 (%) | 25 (50) |
| *BRAF* mutation class, number (%) |  |
| Class I | 13 (25.5) |
| Class II | 20 (39.2) |
| G469 | 13 (25.5) |
| Class III | 11 (21.6) |
| D594 | 7 (13.7) |
| Other | 7 (13.7) |

^*^Table provides median values for each parameter and number of patients and/or percentages of the cohort. For WHO and ICC classifications, categories with more than 1 patient are shown with all others grouped in “Other”.

^†^Among these patients, eight has AML arising from underlying CMML.

**Supplementary Table 2.** Summary of whole exome sequencing (WES) results from 5 *BRAF*-mutant AML patients.^*,†,‡,§^

| **Pt ID** | **Gene** | **Mutation Type** | **VAF Diagnosis** | **VAF Relapse** |
| --- | --- | --- | --- | --- |
| A | *TET2* | FS | 99.88 | NA |
|  | *ATP8B4* | SPL | 51.35 |  |
|  | *ECPAS* | SNV | 48.84 |  |
|  | *STOML2* | SNV | 48.83 |  |
|  | *NPM1* | FS | 48.00 |  |
|  | *NRAS* | SNV | 45.60 |  |
|  | *SRSF2* | SNV | 45.09 |  |
|  | *ABCG5* | SNV | 44.69 |  |
|  | *ARHGAP2* | SNV | 44.57 |  |
|  | *SHANK3* | SNV | 43.09 |  |
|  | *GRIP1* | SNV | 42.88 |  |
|  | ***BRAF*** | **SNV** | **42.28** |  |
|  | *MOV10* | SNV | 39.34 |  |
|  | *ZNF571* | FS | 34.27 |  |
|  | *ZNF850* | SNV | 11.60 |  |
|  | *AQP7* | SNV | 4.43 |  |
|  | *FAT3* | SNV | 4.31 |  |
|  | *RELN* | SPL | 3.25 |  |
| B | *NPM1* | FS | 60.58 | NA |
|  | *KAT6B* | FS | 57.16 |  |
|  | *ANKRD62* | SNV | 52.80 |  |
|  | *RNF213* | SNV | 50.86 |  |
|  | *KRAS* | SNV | 30.26 |  |
|  | *BICDL1* | SNV | 4.40 |  |
|  | *BOP1* | FS | 3.38 |  |
|  | *FAM104A* | FS | 3.19 |  |
|  | *DZIP1* | SNV | 3.05 |  |
|  | *CCDC50* | SPL | 2.90 |  |
|  | *RBM4* | DEL | 2.78 |  |
|  | *MRPS18A* | SNV | 2.19 |  |
|  | ***BRAF*** | **SNV** | **3.00** |  |
| C | *PRUNE2* | SNV | 50.50 | NA |
|  | *MCTP1* | SNV | 49.15 |  |
|  | *ESRP1* | SNV | 48.21 |  |
|  | *CDH23* | SNV | 46.52 |  |
|  | *KRAS* | SNV | 33.62 |  |
|  | *DCDC1* | TR | 25.83 |  |
|  | *DENND2A* | SNV | 22.95 |  |
|  | *NPY2R* | SNV | 18.91 |  |
|  | *ADAMTS1* | SNV | 9.63 |  |
|  | ***BRAF*** | **SNV** | **3.02** |  |
|  | *SP8* | DEL | 2.99 |  |
|  | *BMP2K* | DEL | 2.44 |  |
|  | *CNKSR2* | SNV | 2.26 |  |
|  | *CCDC22* | SNV | 2.14 |  |
| D | *DNAJC11* | SPL | 0.00 | 50.50 |
|  | *NPM1* | FS | 51.81 | 50.00 |
|  | *SCML1* | SNV | 46.30 | 49.76 |
|  | *AKAP11* | SNV | 43.77 | 49.38 |
|  | *NCKAP5* | SNV | 40.66 | 48.68 |
|  | *FBXL15* | SNV | 50.15 | 47.46 |
|  | *TET2^§^* | TR; SNV^§^ | 50.47; 38.51; 0.32 | 46.96; 44.51; 0.0 |
|  | *TUBB8* | SNV | 48.25 | 45.29 |
|  | *ATP13A1* | SNV | 48.76 | 43.45 |
|  | *ACTL8* | SNV | 51.89 | 43.24 |
|  | *SIRT4* | SNV | 46.06 | 43.06 |
|  | *GALK1* | SNV | 48.36 | 42.93 |
|  | *LYN* | SNV | 51.09 | 42.82 |
|  | *KLHL17* | SNV | 43.21 | 42.09 |
|  | *IQGAP3* | SNV | 0.07 | 42.06 |
|  | *ADAMTS1* | SNV | 49.26 | 42.01 |
|  | *NR1H2* | SNV | 40.62 | 40.96 |
|  | *STAG2* | SNV | 36.86 | 40.87 |
|  | *NYX* | SNV | 40.71 | 40.86 |
|  | *DMD* | SNV | 49.58 | 39.69 |
|  | *HNRNPM* | SNV | 43.24 | 39.30 |
|  | *C12orf4* | SNV | 49.27 | 39.28 |
|  | *ATN1* | DEL | 0.00 | 33.08 |
|  | *PUM1* | SNV | 0.00 | 3.54 |
|  | *DNX35* | SPL | 0.00 | 3.10 |
|  | *MAP4* | SPL | 0.39 | 2.79 |
|  | *TRIP4* | SNV | 0.35 | 2.49 |
|  | *SIGLEC16* | SNV | 0.94 | 2.46 |
|  | *SOX1* | SNV | 1.32 | 2.19 |
|  | *FLT3* | SNV | 22.73 | 0.00 |
|  | *PTPN11* | SNV | 17.54 | 0.00 |
|  | ***BRAF*** | **SNV** | **4.55** | **0.00** |
|  | *LAMB4* | SPL | 4.30 | 0.00 |
|  | *WAC* | FS | 3.59 | 0.00 |
| E | *TP53* | SNV | 35.44 | 97.99 |
|  | *LOC10192* | SNV | 50.07 | 53.01 |
|  | *TMPRSS6* | SPL | 32.93 | 51.57 |
|  | *FBXW9* | SNV | 39.27 | 49.92 |
|  | *ASB15* | SNV | 51.79 | 48.88 |
|  | *CHST6* | FS | 51.01 | 47.87 |
|  | ***BRAF*** | **SNV** | **45.79** | **47.76** |
|  | *MROH2B* | SPL | 47.75 | 47.34 |
|  | *NRAS* | SNV | 42.12 | 45.90 |
|  | *NALCN* | SNV | 43.95 | 45.83 |
|  | *STK25* | SNV | 44.58 | 45.28 |
|  | *SLC4A7* | SNV | 42.97 | 45.20 |
|  | *ACLY* | SNV | 45.21 | 44.30 |
|  | *TIMP2* | SNV | 48.98 | 43.91 |
|  | *TJP1* | SNV | 52.52 | 42.68 |
|  | *PNLDC1* | SNV | 45.42 | 41.91 |
|  | *CHD1* | SNV | 42.46 | 41.60 |
|  | *UTRN* | SNV | 50.37 | 37.18 |
|  | *MGA* | SNV | 41.95 | 36.87 |
|  | *POGLUT3* | SPL | 0.00 | 10.66 |
|  | *VGLL4* | SNV | 0.47 | 4.71 |
|  | *ST6GAL1* | SPL | 0.00 | 4.38 |
|  | *SYNE1* | SNV | 0.00 | 4.37 |
|  | *DEAF1* | DEL | 3.17 | 4.32 |
|  | *RELN* | SNV | 0.00 | 3.15 |
|  | *CHRM4* | FS | 0.53 | 2.79 |
|  | *KLF2* | SNV | 0.00 | 2.52 |

^*^Patients A-C only had diagnosis sample available.

^†^Mutated genes listed in descending variant allele frequency (VAF) order in relapse sample or diagnosis sample if relapse sample not available (NA).

^‡^Mutation types are denoted as FS: frameshift, SNV: single nucleotide variant, DEL: non-frameshift deletion, SPL: splice site mutation, and TR: truncation.

^§^Denotes more than 1 mutation or mutation type in same gene.

**Supplementary Fig. S1. A-F)** UMAP (A,C,E) and corresponding violin plots (B,D,F,) of cells from Fig. 2A denoting low (grey) or high (purple) expression of CD14 (**AB**), CD123 (**CD**) or FLT3 (**EF**) with colors in **B**, **D**, and **F** denoted by genotype: WT (grey), *NRAS* (red), *PTPN11* (black), *KRAS* (yellow), *BRAF V600* (dark blue), *BRAF G469* (green), and *BRAF D594* (purple). Each dot within violin plot represents a cell. **G**) Dot plot of row normalized expression of each cell surface protein marker for clones from *BRAF*-mutant samples and clones from *BRAF-*WT*/ RAS*-mutant samples. **HI**) Faceted UMAP from (Fig. 2H) denoting *BRAF*-mutant cells (**H**) and *BRAF-*WT*/RAS*-mutant cells (**I**) with low (grey) or high (red) expression of CD14. **J**) Corresponding violin plot for CD14 expression from cells in **HI**. **KL**) Faceted UMAP from (Fig. 2H) denoting *BRAF*-mutant cells (**K**) and *BRAF-*WT*/RAS*-mutant cells (**L**) with low (grey) or high (red) expression of CD123. **M**) Corresponding violin plot for CD123 expression from cells in **KL.** Asterisks denote *P* <0.05, *; *P*<0.01, **; *P*<0.001, ***, *P*<0.0001, **** (**B,D,F,J,M**).

**Supplementary Fig. S2.** Kaplan-Meyer survival curves of (**A**) all *BRAF*-mutant patients (n=50), (**B**) AML patients high-intensity treated by ELN 2022 status [*BRAF*-mutated (n=14), Favorable (n=23), Intermediate (n=14), Adverse (n=135)], and (**C**) relapse/refractory and secondary AML *BRAF*-mutant patients (n=28) stratified by whether prior treatment regimens had included venetoclax (n=7, blue) compared to not (n=21 purple).

**Supplementary Fig. S3. A)** Volcano plots of the relative sensitivity/resistance of *BRAF* (blue), *NRAS* (red), and *KRAS* (yellow) mutant AML samples to Trametinib (left) and CI-1040 (right) compared to samples from all other *RAS* WT AML genotypes (black). **BCD**) Bar plots denoting area under the curve (AUC) results for *RAS WT* (black), *NRAS*-mutant (red), *KRAS*-mutant (yellow), or *BRAF*-mutant (blue) samples treated with (**B**) Panobinostat (WT: n=227, NRAS: n=52 KRAS: n=12, BRAF: n=5), (**C**) 17-AAG (WT :n=402, NRAS: n=61, KRAS: n=18, BRAF: n=5), or (**D**) AT7519 (WT: n=367, NRAS: n=70, KRAS: n=21; BRAF: n=5). *P* values provided or asterisks denote *P* <0.05, *; *P*<0.01, **; *P*<0.001, ***, *P*<0.0001, **** (**BCD**).
