# Supplementary material for: Prognostic and therapeutic implications of BRAF mutations in acute myeloid leukemia
Source: Leukemia. Author manuscript; Available in PMC 2026 Jul 31. (PMC13421326; doi:10.1038/s41375-026-02996-1)
Supplement: Supplementary Table 3 [file NIHMS2196509-supplement-Supplementary_Table_3.docx]

| **Supplementary Table 3.** Summary of 21 *BRAF*-mutant AML Newly Diagnosed patients | | | | | | | | | | | | | |
| --- | --- | --- | --- | --- | --- | --- | --- | --- | --- | --- | --- | --- | --- |
| **UPIN** | **Sex** | **Age (y)** | **Blasts BM %** | **ICC** | **2022 ELN Risk group** | **Treatment** | **Best response** | **Relapse** | **HSCT** | **Follow-up (mo)** | **Status at last follow-up** | **BRAF, VAF** | **All mutations** |
| 1 | Male | 64 | 35 | AML-MR | Adverse | Aza+Ven+Gilt | MRD- CR | No | Yes | 6 | Alive | p.G469V 1% | BRAF p.G469V;FLT3 p.Q494L; FLT3 p.D835E; FLT3 p.D839G; KRAS p.G12S |
| 7 | Female | 81 | 25 | AML with TP53 | Adverse | DAC+VEN | MRD+ CR | Yes | No | 15 | Dead | p.G469A 2% | BRAF p.G469A; TP53 p.R342*;TP53 p.Y205* |
| 8 | Male | 74 | 20 | AML-MR | Adverse | DAC+VEN | MRD+ CR | Yes | No | 5 | Dead | p.V600E 2% | BCOR p.P305del; BRAF p.V600E; SRSF2 p.P95H; TET2 p.T1393K; TET2 p.Q1526* |
| 10 | Female | 82 | 25 | AML-MR | Adverse | Hydrea +  cytarabine | Persistent disease | NA | No | 3 | Dead | p.V600E 17% | CBL p.Q367P; SRSF2 p.P95L; TET2 p.S842*;TET2 p.Q916* |
| 13 | Female | 59 | 51 | AML with KMT2A-r | Intermediate | Cladribine + LDAC | Persistent disease | NA | No | 1 | Dead | p.V600E 1% | BRAF p.V600E; GATA2 p.G100_G101insFALAGR; GATA2 p.I333fs; IKZF1 p.E16*;KRAS p.G12V |
| 15 | Male | 63 | 78 | AML-MR | Adverse | Aza+Ven+  AG120 | MRD+CR | Yes | No | 32 | Dead | p.G469A 2% | ASXL1 p.G646fs*11;BRAF p.G469A;IDH1 p.R132C;SRSF2 p.P95H;TET2 p.K1439fs;TET2 p.R1543* |
| 17 | Female | 62 | 67 | AML with NPM1 mut | Adverse | CLIA-dexrazoxane and GO - midostaurin | MRD-CR | No | No | 22 | Dead | p.V600E 15% | BRAF p.V600E;DNMT3A p.R882C;FLT3 p.D835H;HNRNPK p.E85K;IDH1 p.R132H;KIT p.D816V;NPM1 p.W288fs;NRAS p.G13D |
| 18 | Female | 74 | 26 | AML with TP53 | Adverse | DAC+Ven | persistent disease | NA | No | 1 | Dead | p.L597R 66% | BRAF p.L597R;RUNX1 p.A398fs;SRSF2 p.P95H;TET2 p.K845*;TP53 p.P222L |
| 21 | Male | 84 | 23 | AML-MR | Adverse | Hydrea | persistent disease | NA | No | 0 | Dead | p.G469E 17% | ASXL1 p.G967del;ASXL2 p.R614*;BRAF p.G469E;CALR p.E398_D400del;KRAS p.A146P;TET2 p.W564fs;TET2 p.V1900F;ZRSR2 p.F174fs |
| 24 | Female | 70 | 50 | AML-MR | Adverse | Dac+Ven | Persistent disease | NA | No | 18 | Dead | p.L597R 2% | ASXL1 p.S846fs;BRAF p.L597R;FLT3 p.I867S;KRAS p.G12D;NRAS p.G12D |
| 28 | Female | 43 | 66 | AML with NPM1 mut | Favorable | ARA-C. | MRD-CR | No | Yes | 91 | Alive | p.V600E 8% | BRAF p.V600E;DNMT3A p.R882H;NPM1 p.W288fs |
| 29 | Male | 56 | 22 | AML-MR | Adverse | FIA | MRD+CR | Yes | No | 35 | Dead | p.G469A 45% | ASXL1 p.G646fs;BRAF p.G469A;TET2 p.R1440fs |
| 34 | Male | 78 | 84 | AML-MR | Adverse | DAC then BIDFA | Persistent disease | NA | No | 5 | Dead | p.L485W 45% | ASXL1 p.E928fs;BRAF p.L485W;IDH1 p.R132C;RUNX1 p.R162G |
| 36 | Male | 71 | 75 | AML-MR | Adverse | Cladribine/ LDAC/ VEN | Persistent disease | NA | No | 2 | Dead | p.V600E 31% | KDM6A p.?;TET2 p.R1440fs*38;TET2 p.Q278*;BRAF p.V600E;CREBBP p.V1801fs*67;CREBBP p.?;NRAS p.G12D |
| 38 | Female | 61 | 60 | AML-MR | Adverse | CPX–351+ venetoclax, | MRD-Cri | Yes | Yes | 4 | Dead | p.V600E 14% | TET2 p.R550*;SRSF2 p.K52M;BRAF p.V600E;SF3B1):c.2098A>G p.K700E |
| 39 | Male | 29 | 73 | AML-MR | Intermediate | 7+3+  midostaurin, then gilt | CRi | No | Yes | 57 | Alive | p.D594G 4% | BRAF p.D594G; FLT3-ITD |
| 40 | Male | 19 | 92 | AML with KMT2A-r | Adverse | 7+3 | MRD- CR | No | Yes | 89 | Alive | p.V600E 4% | BRAF p.V600E; KRAS p.G13D |
| 41 | Female | 27 | 59 | AML with RUNX1::RUNX1T1 | Favorable | 7+3, then MEC+KPT | MRD- CR | No | Yes | 89 | Alive | p.G469A 47% | BRAF p.G469A; TET2 p.S1518fs |
| 42 | Female | 36 | 28 | AML with KMT2A-r | Intermediate | 7+3+  pinometostat | MRD- CR | Yes | No | 6 | Dead | p.D594N 39% | BRAF p.D594N; NRAS p.G12S; TP53 p.Y234C |
| 43 | Female | 67 | 40 | AML with TP53 | Adverse | 7+3 | nonevaluable for response | No | No | 0 | Dead | p.N581S 33% | BRAF p.N581S; KRAS p.T58I; TP53 p.T58I |
| 44 | Male | 67 | 28 | AML with CBFB::MYH11 | Favorable | 7+3 | MRD- CR | Yes | No | 23 | Dead | p.Q494K 51% | BRAF p.Q494K; NRAS p.G12D |
