# Supplementary material for: Prognostic and therapeutic implications of BRAF mutations in acute myeloid leukemia
Source: Leukemia. 2026 Jun 15;40(8):1688–97. doi: 10.1038/s41375-026-02996-1 (PMC13421326; doi:10.1038/s41375-026-02996-1)

Supplemental Figure 1

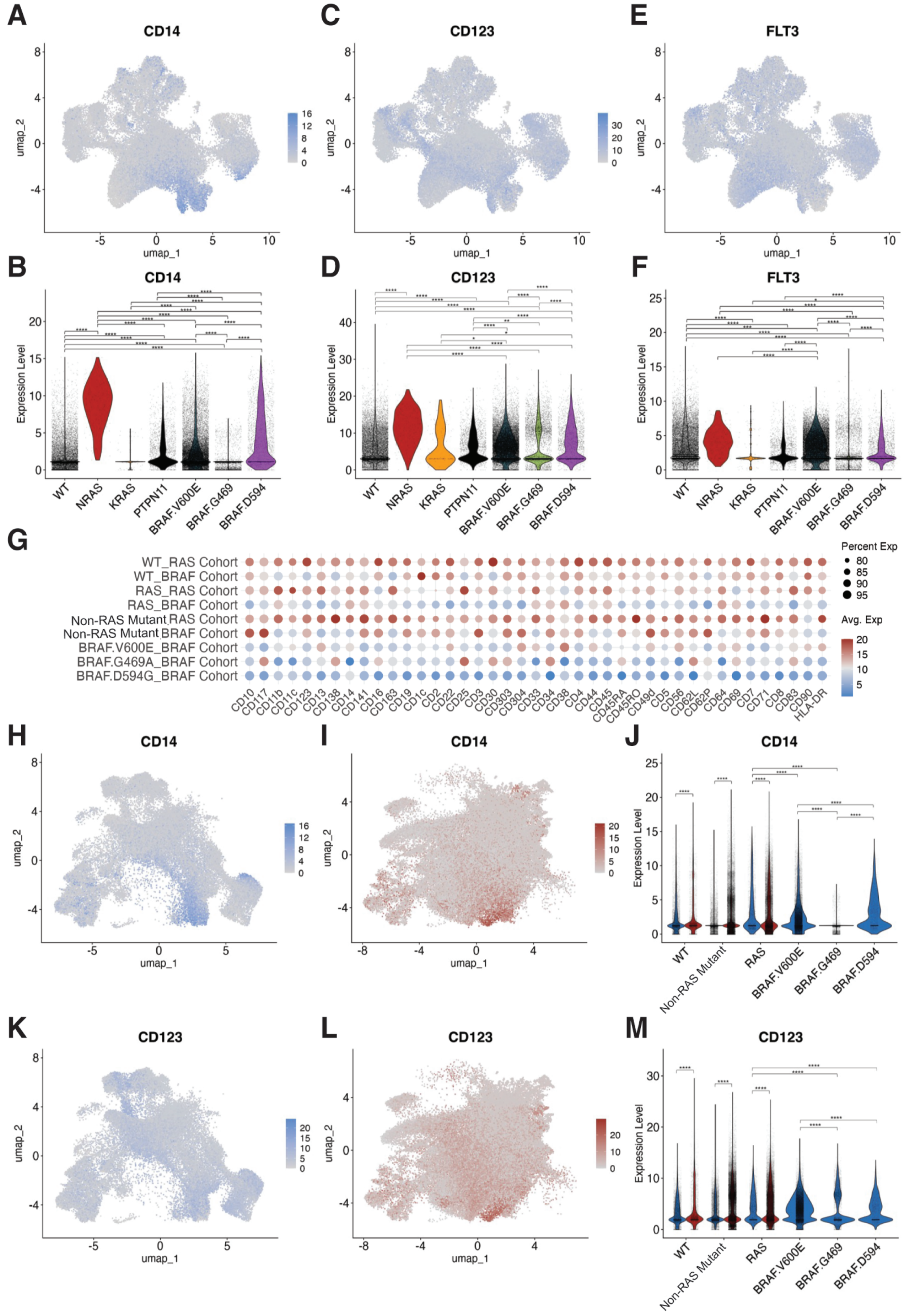

Supplement: Supplementary file 1 [file 41375_2026_2996_MOESM3_ESM.pdf]
